# A comparative assessment of CD4 recovery in a cohort of patients on different HAART regimens in a Nigerian tertiary healthcare facility

**DOI:** 10.4314/ahs.v24i2.3

**Published:** 2024-06

**Authors:** Paul Onah, Catherine Idoko, Aliyu Kai'gama, Siyaka Abdulateef

**Affiliations:** 1 University of Maiduguri Bama Road, Maiduguri, Borno State, Maiduguri 600230 Nigeria; 2 University of Benin, Clinical pharmacy and pharmacy practice; 3 University of Maiduguri, Department of Clinical Pharmacy and Pharmacy administration

**Keywords:** CD4 recovery, different HAART regimens, Nigerian tertiary healthcare facility

## Abstract

**Background:**

Antiretroviral therapy is expected to produce sustained viral load reduction and a rise in CD4 cell count, both of which are important clinical markers of immune recovery. There is contrasting clinical evidence of CD4 stability among patients on long term therapy, which is a major challenge in poor resource settings. This study aims to evaluate CD4 cell recovery among patients on four regimens who have been on long term antiretroviral therapy

**Methods:**

This was a retrospective cohort study using data from the medical records of patients on four antiretroviral regimens. A three year record of CD4 cell count of 405 randomly selected subjects was extracted for analysis.

**Results:**

The increase of CD4 cells was between 65.6 – 82.1% of baseline values, with the highest rise occurring with Efavirenz based regimens. Among patients who achieved target CD4 cell counts ≥ 500 cells/ml, there was further increase of between 22.2 – 34.1% compared to baseline values. The percentage of patients with incomplete immune recovery still remain high among patients on the four regimens 65.9 – 77.8%.

**Conclusion:**

Immune reconstitution continue to occur among patients, however a significant proportion of patients fail to achieve and sustain target CD4 target on the long term.

## Introduction

One of the major clinical markers of immune recovery among patients on highly active antiretroviral therapies HAART is the improvement in CD4 T lymphocyte count. It is considered to be an important predictor of disease progression, occurrence of opportunistic infections and long term reconstitution of immune function in patients living with HIV infection 1,2 It is expected that patients on HAART will achieve normal CD4 count and undetectable viral load both of which are indicated for monitoring disease progression. Immune reconstitution following antiretroviral therapy occur in three distinct phases all of which can be explained by the rate of CD4 recovery.

In the first phase, which usually last for 2 – 3 months of therapy, the number of peripheral CD4 cells increase rapidly at the rate of 20 – 30 cells/mm^3^/month^3^,^4^,^5^. The second phase of recovery generally slow down to between 5 – 10 cells/mm^3^/month, while the third phase is characterized by even slower increases of between 2 – 5 cells/mm^3^/month. The last two phases involves de novo production of CD4 cells by the thymus gland, homeostatic proliferation and extension of the half-life of circulating CD4 cells.

Many patients who achieved effective viriologic suppression, often fail to optimize and sustain CD4 recovery over long periods of therapy. A significant percentage of patients on therapy not only fail of achieve optimal immune recovery, but those who do may take years to have normal CD4 cell count^6^,^7^. A number of studies reported that up to a third of patients on therapy do not achieve normal CD4 cell count within five years of therapy^8^,^9^,^10^. The recovery of CD4 positively correlates with lower risk of opportunistic infections, disease progression and mortality^11^,^12^.

The long term effect of HAART on CD4 cell recovery is a subject of debate because questions still remain as to whether or not the initial CD4 cell rise is maintained over long period of time 13. While some studies reported that CD4 cell rise following initiation of therapy vary widely between antiretroviral regimens, this has not been consistently observed between patients^3^, ^14^. A number of comparative studies have reported that CD4 cell recovery differ widely among patients on different regimens^15^,^16^,^17^,^18^,^19^. For instance, some studies reported better CD4 recovery rates with Efavirenz containing regimens^15^,^20^,^21^, while others reported contrasting recovery rates^13^.

Several longitudinal studies carried out in north American and European adults living with HIV showed that CD4 recovery continued to improve after 4 – 7 years of therapy^22^,^23^,^24^,^25^ and stabilized^26^,^27^ or at least remained stable in a subset of patients^28^. The trajectory of CD4 recovery is largely unknown largely due to differences in patient specific characteristics, CD4 at initiation of therapy^29^, presence of co-infections, treatment interruptions and adherence, all of which may explain the highly variable study conclusions.

A review of randomized clinical trials involving Nevirapine and Efavirenz based regimens have demonstrated good CD4 recovery rates, though the latter has been reported as a better choice as initial therapy^13^. In contrast, a number of other studies reported short term increase in CD4 recovery with all regimens^30^, however there was no significant difference in the rate of immune reconstitution on the long term.

Some patients who failed to achieve normal CD4 counts after long term therapy ≥ 7 years have been reported to have had low CD4 at the point of initiating therapy 30,31, though this observation may not be representative of all subset of patients.

A number of determinants of immune recovery included increasing age 32,33,34,35, viral suppression^36^,^37^, CD4 count at initiation of therapy 38, the presence and severity of co-morbidities.

The achievement and stability of normal CD4 count over long period of time is critical to optimal immune recovery, improved quality of life, lower risk of opportunistic infections and risk of mortality. The aim of this study is to assess CD4 cell recovery among patient receiving different HAART regimens

## Methods

**Setting:** The study was carried out at the ARV clinic of University of Maiduguri teaching hospital. This is a public tertiary hospital which also serve as a referral centre for both private and public health facilities in the Nigeria's north east region. The HIV clinic provide services which include diagnosis, treatment and prevention as well as management of complications and comorbidities. The clinic units included adult ART unit, paediatric ART unit, prevention of mother to child transmission PMTCT unit and laboratory.

### Study design

This was a cross sectional retrospective study using data obtained from the medical records of patients who have been on HAART regimens for at least two years and above at the start of the study. The three year CD4 records obtained were from 2016, 2017 and 2018.

**Sample size:** A total 405 eligible subjects were selected and records used for the study. The medical records were separated according to their HAART regimen before selection by simple random method.

**Eligibility:** The subjects must have been above 18 years and have been on HAART for at least two years at the time of the study. They must have at least attended 90% of physician appointment and have routine CD4 test done at least once a year during the study period.

**Data collection:** Data was extracted from medical and laboratory records as related to CD4 counts during the three year study period 2016 – 2017. The primary outcome measure for complete CD4 recovery was ≥ 500 cells/ml.

### HAART regimens

#### Regimen I

Tenofovir/Lamivudine/Efavirenz (n = 266)

Regimen II Zidovudine/Lamivudine/Nevirapine (n=77)

Regimen III Tenofovir/Lamivudine/Dolutegravir (n=44)

Regimen IV Zidovudine/Lamivudine/Lopinavir/Ritonavir (n=18)

### Data analysis

The data was double checked for accuracy and entered into SPSS 21 for descriptive and inferential statistics. The analysis was carried out using one way ANOVA Tukey post Hoc and P values ≤ 0.05 was considered statistically significant. The proportion of subjects with incomplete CD4 recovery was expressed using descriptive statistics. Ethical approval: The health research ethics committee of the University of Maiduguri teaching hospital approved the study.

## Results

The results showed that the population of females almost doubled that of males. The mean age of patients was 35.410.2 years and majority of subjects had primary level education (69.1%) and were married (69.4%) Table 1.

**Table 1:** Demographic data

Variable	Number %
**Gender**	
Male	177 35.4
Female	324 64.6
**Education**	
Illiterate	305 61
Primary	75 15
Secondary	88 17.6
Tertiary	32 6.4
**Marital status**	
Single	52 10.4
Married	347 69.4
Divorced	78 15.6
Widowed	23 4.6
**Occupation**	
Self employed	184 36.8
Civil service	85 17
Unemployed	210 42
Student	21 4.2
Mean age yrs.	**35.4 ± 10.2**
Duration on HAART yrs.	
1 – 3	100 24.6
4 – 6	221 54.6
7 – 9	51 12.6
≥ 10	33 8.2
Mean SD	**5.1 ± 2.5**

There was statistically significant increase in CD4 cell count with the four HAART regimens. The rise was about 82.1% among patients on regimen I, 66.8% regimen II, 70.9% regimen III and 65.6% in patients on regimen IV during the three year study period Table 2.

**Table 2:** Comparison of CD4 cell count among patients on different HAART regimens

	Year 2016	Year 2017	Year 2018	P value	CD4 increase %
Regimen I *n=266*	365.5 161.2	466.1 158.2	665.5 162.8	<0.0001	82.1
Regimen II *n=77*	380.5 183.3	479.2 153.4	634.5 182.3	<0.0001	66.8
Regimen III *n=44*	408.9 171.3	468.7 130.1	698.9 211.7	<0.0001	70.9
Regimen IV *n=18*	379.8 157.4	428.5 116.5	629.1 220.8	<0.0001	65.6

Among patients who achieved immune recovery at the beginning of the study ≥ 500 cells/ml, there was also a significant increase in CD4 cell count except for patients on regimen IV. The overall increase in this subset was between 22.2 – 34.1% of baseline values. Table 3.

**Table 3:** Comparison of CD4 cell count among patients with immune recovery ≥ 500 cells/ml

	Year 2016	Year 2017	Year 2018	P value	CD4 increase %
Regimen I *n=66*	594.3 87.2	675.4 102.7	618.9 94.8	<0.0001	24.8
Regimen II *n=20*	568.2 48.6	611.7 66.9	657.1 72.9	0.0002	25.9
Regimen III *n=15*	533.7 20.8	597.8 37.8	689.5 50.7	<0.0001	34.1
Regimen IV *n=4*	574.4 61.8	609.6 41.5	653.7 80.4	0.2585	22.2

A comparison of CD4 cell count showed significant differences between the four regimens < 0.001. This result showed that in spite of therapy many patients continued to have poor immune recovery irrespective of HAART regimen. Patients with CD4 cell counts of ≤ 200 cells/ml are particularly at a higher risk of opportunistic infections and disease progression Table 4

**Table 4:** Comparison of CD4 cell increase between HAART regimens

CD4 count	Regimen I *n=266*	Regimen II *n=77*	Regimen III *n=44*	Regimen IV *n=18*	P value
≤ 100	80.7 ± 5.2	72.7 ± 12.1	na	92.1 ± 2.7	< 0.001
101 – 200	179.2 ± 20.3	189.1 ± 7.5	154.4 ± 9.9	166.2 ± 10.4	< 0.001
201 - 300	287.9 ± 9.1	256.3 ± 22.7	275.5 ± 15.7	255.5 ± 13.9	< 0.001
301 – 400	365.5 ± 31.1	380.5 ± 13.3	348.9 ± 22.2	379.8 ± 11.1	< 0.001
401 – 500	466.1 ± 18.2	477.2 ± 20.2	450.2 ± 10.1	447.2 ± 16.7	< 0.001
501 - 600	532.5 ± 10.8	535.5 ± 19.4	575.6 ± 17.2	581.7 ± 12.5	< 0.001
≥ 601	798.5 ± 55.6	733.5 ± 8.7	822.2 ± 60.7	676.5 ± 27.8	< 0.001

**Figure 1:**
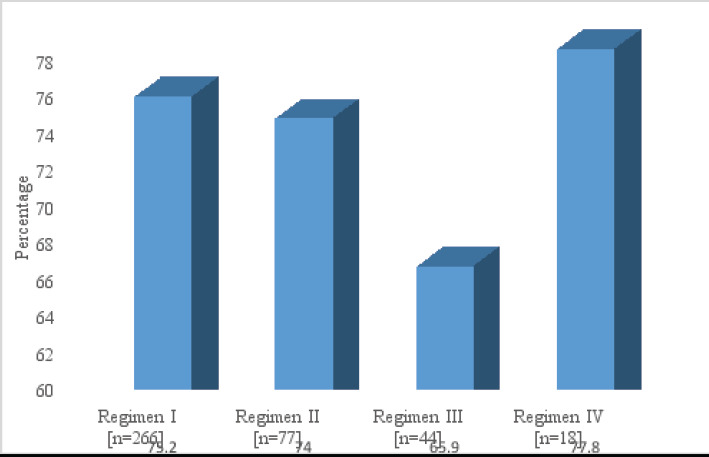
Distribution of patients with incomplete immune recovery ≤ 500 cells/ml

The results showed a high percentage of patients with incomplete immune recovery <500 cells/ml with the four regimens. The distribution showed that between 65.9 – 77.8% of all patients did not achieve target CD4 cell count during the study period Figure 1.

## Discussion

Antiretroviral therapy is known to not only improve CD4 count and immune recovery, it also reduces viral load and improve patient's quality of life. The rise of CD4 T-cell lymphocyte count following initiation of therapy has been used as a clinical marker of immune recovery among patients living with HIV. Majority of subjects in this study were females which was comparable to some previous studies^39^,^40^,^41^, which is reflective of the fact that women in sub Saharan Africa are at higher risk of infection compared to males.

The CD4 cell count significantly fluctuated among patients on the same regimen. Among patient who achieved target CD4 recovery the variation was also significant. While the percentage of patients who failed to achieve complete immune reconstitution was high like in some previous studies^42^,^43^,^44^, the increase in CD4 occurred though at significantly different rates^45^,^46^.

The results clearly showed that CD4 recovery was still ongoing, a significant percentage of patients did not achieve targets 47. There is clinical evidence to believe that immune restoration with CD4 cells levels of ≥ 500 cells/mm^3^ is associated with comparable mortality rates with uninfected individuals, so this cutoff value has been widely used to benchmark immune reconstitution in many studies^48^,^49^ similar to this study.

The increase of CD4 cell observed across the four regimens was comparable to earlier previous studies^50^,^51^. The percentage of patients with incomplete CD4 recovery is considerably higher than 20 – 49.7% reported^7^,^52^,^53^. Patients are expected to achieve and sustain immune recovery early in the course of therapy, studies have established that many patients still fail to achieve normal CD4 cells, though the rate in this study is comparatively higher 9. A number of other studies however reported contrasting levels of immune recovery^8^,^54^, which suggests that achieving optimal CD4 recovery is far from being a certain clinical outcome^55^, ^56^, ^57^

The rise in CD4 is known to be nonlinear and unrelated to HAART regimen which indicated no demonstrable superiority exist between regimens^55^,^58^. There is some literature evidence that Tenofovir based regimens were better than Zidovudine based regimens^59^,^60^, though contrasting conclusions have been reported in one study^61^.

Similar studies reported no differences in CD4 recovery between Nevirapine and Efavirenz based regimens 21, however other studies appear to contradict this conclusion 36. The highly variable effect of long term HAART on CD4 cell recovery shown by several studies^62^,^63^, ^64^; indicated a number of determining factors some of which include, duration of therapy 13, 28, low CD4 cell at initiation of therapy 24, viral suppression, advancing age, non-adherence, haemoglobin level, adverse drug reactions^39^,^45^,^46^.

The low CD4 recovery may also be associated with poor viral suppression and extensive immunological damage as reported in some studies^65^. This non-optimization of immune recovery among patients on long term therapy significantly increase the frequency and severity of opportunistic infections^66^ and ultimately poor clinical outcomes.

## Conclusion

This study clearly showed that immune reconstitution continue to occur among patients on all regimens, however a significant percentage of them did not achieve target CD4 cell count. There is need to strengthen routine monitoring as incomplete immune recovery still remain a long term challenge to the achievement of positive clinical outcomes.
